# Genetic, Socioecological, and Health Determinants of Extreme Longevity in Semi-Supercentenarians and Supercentenarians: Protocol for a Scoping Review

**DOI:** 10.2196/63900

**Published:** 2025-03-05

**Authors:** Wafa Abu El Kheir-Mataria, Omnia Mahmoud Abdelraheem, Sungsoo Chun

**Affiliations:** 1 Institute of Global Health and Human Ecology School of Science and Engineering American University in Cairo New Cairo Egypt

**Keywords:** supercentenarians, semi-supercentenarians, extreme longevity, genetic factors, socioecological factors, health determinants, aging research, scoping review, cognitive performance, data collection methods

## Abstract

**Background:**

The study of supercentenarians (individuals aged 110 years or older) offers valuable insights into aging, longevity, and the factors contributing to exceptional lifespans. These individuals often exhibit extraordinary cognitive and physical performance, which can inform strategies to improve the health of the general population. Research on centenarians (individuals aged 100 years or older), semi-supercentenarians (individuals aged 105-109 years), and supercentenarians covers themes like genetic factors, microbiome, inflammation, diet, lifestyle, and psychological aspects. These studies often focus on various aspects of extreme longevity, using varied objectives and methodologies, highlighting the need for a comprehensive synthesis to map the breadth of research and identify gaps in understanding this demographic.

**Objective:**

This scoping review aims to map and synthesize existing evidence on the determinants of extreme longevity, focusing on individuals living beyond 105 years. This review seeks to categorize genetic factors associated with semi-supercentenarians and supercentenarians; explore the range of socioecological factors contributing to their longevity; and identify common themes such as health, functional capacity, cognition, mental health, behaviors, social support, quality of life, personality traits, environmental factors, and religiosity. Additionally, it aims to examine and describe the methodologies and assessment tools used in studies on extreme longevity and provide an overview of global demographic trends and patterns among supercentenarians, including geographic distribution, gender prevalence, and socioeconomic characteristics.

**Methods:**

This scoping review follows the PRISMA-P (Preferred Reporting Items for Systematic Review and Meta-Analysis Protocols) 2015 guidelines and the Population, Exposure, and Outcome framework. It includes observational and interventional, quantitative and qualitative studies on supercentenarians and semi-supercentenarians. Data will be sourced from databases like Scopus, PubMed, ProQuest, PsycINFO, and The Cochrane Library. The selection process involves abstract and full-text screening by two independent reviewers, with data extraction focusing on study characteristics, participant demographics, interventions or exposures, and key findings. A thematic analysis will identify patterns across various themes

**Results:**

As of October 2, 2024, five databases were searched, yielding 844 studies. After removing duplicates, 706 studies remained. Following the first and second screening stages, 135 studies were found to be eligible. The study is expected to be completed by the end of February 2025.

**Conclusions:**

By synthesizing evidence, this study will understand the global scope of supercentenarians, describe the main themes of research interest, and identify gaps. The findings are expected to contribute significantly to the body of knowledge on longevity, informing future research and public health policies. This scoping review aims to enhance the understanding of factors promoting healthy aging and extreme longevity, benefiting broader public health initiatives.

**Trial Registration:**

PROSPERO CRD42024512298; https://tinyurl.com/4cmux7h4

## Introduction

The study of supercentenarians—individuals who have reached the remarkable age of 110 years or older—holds immense scientific interest. These exceptional individuals provide valuable insights into aging, longevity, and the factors contributing to their remarkable lifespans. Limiting illness—both in duration and the number of individuals affected—becomes more critical as human longevity increases. Studying healthy humans with exceptional lifespans is meaningful in discovering clues to improve the general population’s health. Centenarians (individuals aged 100 years or older), semi-supercentenarians (individuals aged 105-109 years), and supercentenarians (individuals aged 110 years or older) are excellent models for the study of healthy longevity. According to research, this aged population surprisingly maintain extraordinary cognitive and physical performance [1].

These studies provide valuable insights into various aspects of supercentenarian aging and use a variety of methodological frameworks for evaluating the determinants of extreme longevity. Currently, there exists a wide range of studies that investigate exceptional lifespans from diverse perspectives. Sebastiani et al [2] discussed genome sequencing in supercentenarians, revealing common and rare genetic variants that may contribute to exceptional longevity. Franzke et al [3] looked into “DNAging” and discovered that supercentenarians exhibit improved DNA repair and antioxidant defense mechanisms compared to younger cohorts. Santos-Lozano et al [4] discovered that both genetic and environmental factors are attributed to exceptional longevity in centenarians and supercentenarians. Some studies discussed morbidity in supercentenarians and found that cognitive and physical function decline is delayed in supercentenarians [5], while other studies investigated other factors contributing to super longevity in supercentenarians such as diet and lifestyle [6,7].

This scoping review aims to comprehensively synthesize the existing evidence to understand the determinants that contribute to extreme longevity in individuals living beyond 105 years, as well as the methodologies used for data collection.

This scoping review’s main objectives are as follows:

1. To map and categorize the existing evidence on genetic factors associated with extreme longevity in semi-supercentenarians and supercentenarians;
2. To explore and synthesize the range of socioecological factors, including lifestyle behaviors, environmental exposures, and social support systems, that are associated with achieving extreme longevity;
3. To examine and describe the methodologies and assessment tools used in studies investigating extreme longevity; and
4. To provide an overview of global demographic trends and patterns among supercentenarians, including geographic distribution, gender prevalence, and socioeconomic characteristics.

## Methods

### Approach

This scoping review follows the Population, Exposure, and Outcome framework [8] and the PRISMA-P (Preferred Reporting Items for Systematic Review and Meta-Analysis Protocols) guidelines published in 2015 (Multimedia Appendix 1) [9]. The population and outcome are as follows:

- Population: Semi-supercentenarians (aged 105 years or older)
- Exposure: Genetics and socioecological factors
- Outcome: Supercentenarian (aged 110 years or older)

### Eligibility Criteria

#### Study Characteristics

This scoping review will include quantitative and qualitative studies in which supercentenarians and semi-supercentenarians are the focus of the study, including observational research (case-report, case-series, cross-sectional, case-control, and cohort studies) and interventional research (quasi-experimental studies and randomized controlled trials).

#### Types of Participants

This scoping review will target studies in which groups or subgroups of participants comprise male or female individuals aged 105 years and older.

The exclusion criteria will be studies involving participants younger than 105 years as the primary focus.

#### Setting and Time Frame

In this scoping review, articles will be screened initially without any time restrictions. Additionally, there will be no limitations on the study settings.

#### Report Characteristics

Only peer-reviewed studies with full-text availability in English will be included. There will be no restrictions on the date of acceptance or publication. Regarding publication status, only articles that are either published or in press will be considered.

The exclusion criteria will be articles not available in full text, non–English-language studies, and non–peer-reviewed studies (eg, gray literature)

### Information Sources

Our sources of information will be limited to electronic databases. An electronic search will be performed through Scopus (including Scopus Secondary literature), PubMed, ProQuest, PsycINFO, and The Cochrane Library (Cochrane Database of Systematic Reviews, Cochrane CENTRAL, and Cochrane Methodology Register). Additionally, the reference lists of primary studies included in the review and the reference lists of relevant, previously published reviews will be reviewed.

#### Search Strategy

The search syntax used in the different databases is shown in Table 1.

**Table 1:** Search syntax.

Database	Search query	Filters
Scopus Articles	TITLE-ABS-KEY ( *supercentenarian* OR semi*supercentenarian ) AND ( LIMIT-TO ( DOCTYPE , “ar” ) OR LIMIT-TO ( DOCTYPE , “no” ) OR LIMIT-TO ( DOCTYPE , “cp” ) OR LIMIT-TO ( DOCTYPE , “sh” ) OR LIMIT-TO ( DOCTYPE , “le” ) OR LIMIT-TO ( DOCTYPE , “ed” ) )	Articles, notes, conference papers, letters, and editorials; excluded reviews and book chapters
PubMed	“supercentenarian”[Title/Abstract] OR “semi*supercentenarian”[Title/Abstract] OR ((“Centenarians”[MeSH^a^ Terms] OR “Centenarians”[All Fields] OR “centenarian”[All Fields]) AND “Centenarians”[MeSH Terms])	None
ProQuest	(supercentenarian* OR semi*supercentenarian supercenten*arian OR semi*supercentenarian) NOT (at.exact(“Literature Review” OR “Review”) AND PEER(yes)) Ending truncation: Search query: supercentenarian* OR semi*supercentenarian Middle truncation: Search query: supercenten*arian OR semi*supercentenarian Databases: Coronavirus Research Database ProQuest Dissertations & Theses Global Publicly Available Content Database	Peer-reviewed studies; excluded literature reviews and reviews
PsycINFO	*supercentenarian* OR semi*supercentenarian	None
Scopus Secondary Literature (a secondary document is a document extracted from a Scopus reference list, but is not indexed by, or available in, Scopus)	TITLE-ABS-KEY ( *supercentenarian* OR semi*supercentenarian )	None
Scopus Patents	TITLE-ABS-KEY ( *supercentenarian* OR semi*supercentenarian )	None
Cochrane Database of Systematic Reviews	(centenarian):ti,ab,kw OR (supercentenarian):ti,ab,kw AND (semi-supercentenarian):ti,ab,kw	None

^a^MESH: Medical Subject Headings.

Details of the search records, such as the date of the search, database, keywords, number of studies identified, and the number of eligible studies, will be appropriately documented. The authors will follow the adapted PRISMA-P guidelines to report the screening results.

#### Study Records: Selection Process

The results from the database search will be entered into Zotero (Corporation for Digital Scholarship), where duplicate records will be removed. Abstract and full-text screenings of the studies will be conducted by two independent reviewers using the eligibility criteria as a guide. Any disagreements among reviewers following abstract screening will be resolved through discussions to reach a consensus. However, a third reviewer will be involved to address discrepancies at the full-text screening stage.

### Data Extraction and Coding

Data extraction will be done through systematic categorization of information from the included studies. The information will be categorized into predefined fields to ensure uniformity and consistency. The extracted data will include the following categories:

- Study details: Title, author(s), year of publication, journal, and geographic location
- Study design: Observational (eg, case-report, case-series, cross-sectional, case-control, and cohort studies) or interventional (eg, quasi-experimental studies and randomized controlled trials) studies
- Study population: Demographics (age, gender, and socioeconomic background) and sample size
- Factors analyzed: Genetic determinants, socioecological factors (eg, lifestyle behaviors, environmental influences, and social support), and health outcomes
- Methodological details: Data collection methods, assessment tools, and analytical approaches
- Type of data: Quantitative, qualitative, or mixed methods

The extracted data will then be coded and classified into themes based on the study’s objectives and reported findings. Uniformity will be maintained by using a standardized data extraction template and regular discussions among reviewers to resolve ambiguities and discrepancies during the coding process.

### Data Synthesis

Thematic analysis will be conducted to synthesize findings from included studies, following the 6-phase framework by Braun et al [10]. This process includes familiarizing with the data, generating codes, identifying and reviewing themes, and producing the final synthesis. Themes will focus on age validation, demographics, health, functional capacity, cognition, behaviors, social support, quality of life, personality traits, environmental and genetic factors, and religiosity. Themes will be identified inductively to allow for novel insights while remaining aligned with the review objectives.

Reflexivity will be ensured through regular team discussions and documentation of assumptions to minimize researcher bias. Confirmability will be achieved by using two independent reviewers for coding, with discrepancies resolved through discussion or a third reviewer. Dependability will be ensured through a standardized coding process and team reviews of the thematic framework.

Credibility will be supported by providing detailed descriptions of themes. Transferability will be addressed by detailing the contexts and characteristics of the included studies and discussing broader implications for aging research.

## Results

**Figure 1:**
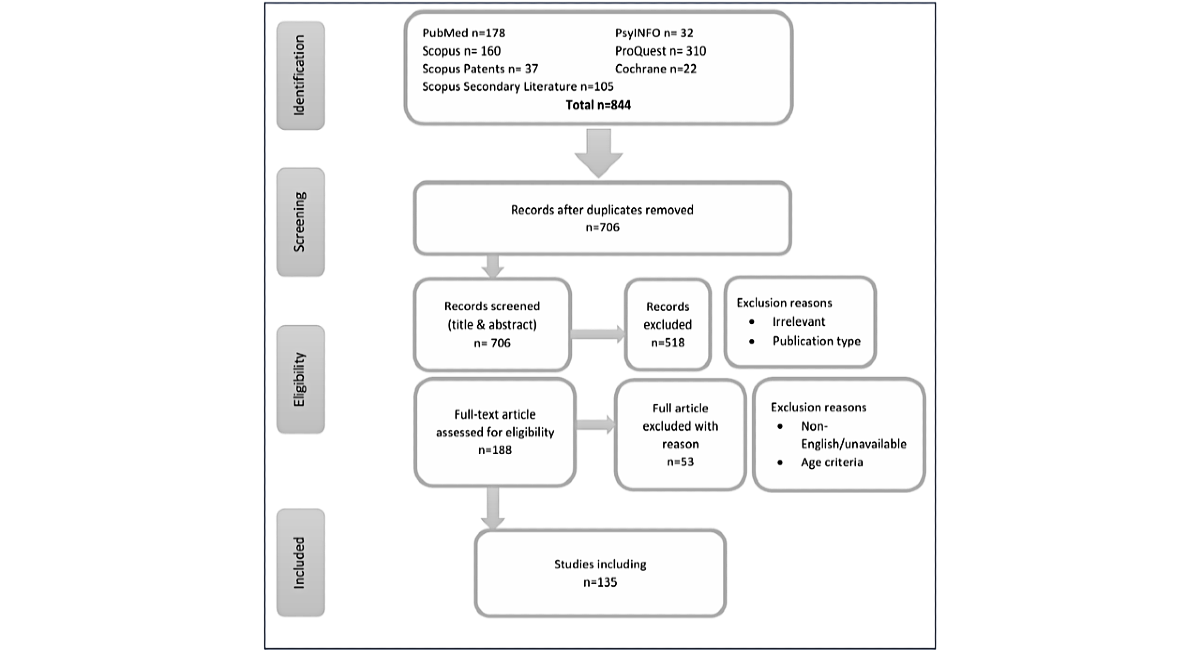
PRISMA (Preferred Reporting Items for Systematic Reviews and Meta-Analyses) flow chart.

As of October 2, 2024, five databases were searched, and 844 studies were retrieved. After eliminating duplicates, 706 studies remained. Following the first stage of title and abstract screening, 518 studies were excluded and 188 studies remained. The remaining studies were subjected to full-text screening against the eligibility criteria, which resulted in eliminating 53 studies, leaving 135 studies to be included (Figure 1). The study is expected to be completed by the end of February 2025.

## Discussion

### Expected Findings

The study of semi-supercentenarians and supercentenarians is extremely valuable in shedding light on healthy aging and longevity [11]. Scholars have studied various areas and numerous factors suspected to contribute to extreme longevity. A study on Italian centenarians and supercentenarians used a multidimensional approach (genetic, demographic, and phenotypic characteristics) to better understand the complex interactions underlying longevity [12]. Other research concentrated on cognition and dementia. Recent research has identified the transcription factor *REST* as an important factor in extreme longevity and cognitive activity [13], while others found that supercentenarians show particularly mild neuropathological findings and thus exhibit remarkable resilience to age-related cognitive decline and dementia [14]. Genetic factors were also studied. A study found strong genetic components to longevity, with siblings of centenarians having significantly higher chances of reaching 90 years [15]. Psychosocial factors, including demographics, personality, and socioeconomic resources, were also found to significantly impact the health and quality of life of centenarians [16]. These are a few studies among many that contribute to the broader discussions on aging theories. On the other hand, more than 25 landmark centenarian and supercentenarian studies have been conducted by specialized organizations across different geographical regions, using diverse methodologies and data collection approaches [17-21]. Given the extensive research on semi-supercentenarians and supercentenarians, this scoping review will serve as a valuable resource for mapping the genetic and socioecological factors associated with individuals aged 105 and older. It will also identify and summarize the methods and assessment tools used to study the key factors contributing to extreme longevity.

A total of 135 studies has been included, reflecting a diverse body of research. However, the wide range of topics, methodologies, and findings highlights the need for a comprehensive synthesis to consolidate existing evidence and identify gaps for future investigation. This review provides an overview of current research on extreme longevity while pinpointing areas that require further study. This scoping review sets the foundation for future standardized research protocols in longevity studies. Additionally, the findings of this study will have important implications for aging-related health care strategies and interventions. Understanding the genetic and socioecological determinants of extreme longevity can lead to personalized health interventions that aim at promoting healthy aging. Policy makers and health care providers can use this evidence to design lifelong healthy behaviors programs and improve social and community support systems for older adults. The findings of this study will contribute to the body of literature on semi-supercentenarians and supercentenarians, inform future studies and interventions, and guide policy makers in the field of aging and public health.

### Study Limitations

This scoping review has limitations that need to be acknowledged. There is a risk of selection bias. This scoping review only includes peer-reviewed, full-text articles in English, which may narrow the findings by excluding relevant studies published in other languages or in gray literature. Moreover, there is difficulty in making direct comparisons due to significant methodological differences, particularly in how longevity determinants are defined and the assessment tools used. Lastly, this scoping review as a study does not establish causal relationships between genetic, socioecological factors, and extreme longevity. Instead, it serves as a foundation by identifying gaps, trends, and inconsistencies in the existing research. It provides insights that can guide future longitudinal studies (which track individuals over time) and experimental research (which tests specific interventions) to better understand the causal mechanisms behind extreme longevity.

## Appendix

Multimedia Appendix 1 PRISMA-P (Preferred Reporting Items for Systematic Review and Meta-Analysis Protocols) checklist.

## Abbreviations

PRISMA-P: Preferred Reporting Items for Systematic Review and Meta-Analysis Protocols

## Data Availability

Data are available upon request from the authors.

## References

[1] Komaki S, Nagata M, Arai E, Otomo R, Ono K, Abe Y, Ohmomo H, Umekage S, Shinozaki NO, Hachiya T, Sutoh Y, Otsuka-Yamasaki Y, Arai Y, Hirose N, Yoneyama A, Okano H, Sasaki M, Kanai Y, Shimizu A (2023). Epigenetic profile of Japanese supercentenarians: a cross-sectional study. Lancet Healthy Longev.

[2] Sebastiani P, Riva A, Montano M, Pham P, Torkamani A, Scherba E, Benson G, Milton JN, Baldwin CT, Andersen S, Schork NJ, Steinberg MH, Perls TT (2011). Whole genome sequences of a male and female supercentenarian, ages greater than 114 years. Front Genet.

[3] Franzke B, Neubauer O, Wagner K (2015). Super DNAging-new insights into DNA integrity, genome stability and telomeres in the oldest old. Mutat Res Rev Mutat Res.

[4] Santos-Lozano A, Santamarina A, Pareja-Galeano H, Sanchis-Gomar F, Fiuza-Luces C, Cristi-Montero C, Bernal-Pino A, Lucia A, Garatachea N (2016). The genetics of exceptional longevity: insights from centenarians. Maturitas.

[5] Andersen SL, Sebastiani P, Dworkis DA, Feldman L, Perls TT (2012). Health span approximates life span among many supercentenarians: compression of morbidity at the approximate limit of life span. J Gerontol A Biol Sci Med Sci.

[6] Newton JP (2007). The supercentenarians and how to get the most out of life. Gerodontology.

[7] Gu Q, Sable CM, Brooks-Wilson A, Murphy RA (2020). Dietary patterns in the healthy oldest old in the Healthy Aging Study and the Canadian Longitudinal Study of Aging: a cohort study. BMC Geriatr.

[8] Mezaoui H, Gunasekara I, Gontcharov A (2019). Enhancing PIO element detection in medical text using contextualized embedding.

[9] Moher D, Shamseer L, Clarke M, Ghersi D, Liberati A, Petticrew M, Shekelle P, Stewart LA, PRISMA-P Group (2015). Preferred Reporting Items for Systematic Review and Meta-Analysis Protocols (PRISMA-P) 2015 statement. Syst Rev.

[10] Braun V, Clarke V, Hayfield N, Davey L, Jenkinson E, Bager-Charleson S, McBeath A (2022). Doing reflexive thematic analysis. Supporting Research in Counselling and Psychotherapy.

[11] Leslie M (2008). Aging. Searching for the secrets of the super old. Science.

[12] Montesanto A, de Rango F, Pirazzini C, Guidarelli G, Domma F, Franceschi C, Passarino G (2017). Demographic, genetic and phenotypic characteristics of centenarians in Italy: focus on gender differences. Mech Ageing Dev.

[13] Marcos-Pérez D, Saenz-Antoñanzas A, Matheu A (2021). Centenarians as models of healthy aging: example of REST. Ageing Res Rev.

[14] Arai Y (2017). The prevalence and risk factors of dementia in centenarians [Article in Japanese]. Brain Nerve.

[15] Willcox DC, Willcox BJ, Hsueh W, Suzuki M (2006). Genetic determinants of exceptional human longevity: insights from the Okinawa Centenarian Study. Age (Dordr).

[16] Poon LW, Martin P, Bishop A, Cho J, da Rosa G, Deshpande N, Hensley R, Macdonald M, Margrett J, Randall GK, Woodard JL, Miller LS (2010). Understanding centenarians' psychosocial dynamics and their contributions to health and quality of life. Curr Gerontol Geriatr Res.

[17] Caselli G, Battaglini M, Capacci G (2022). Italian centenarians and semi-supercentenarians surveys. Encyclopedia of Gerontology and Population Aging.

[18] Young RD (2017). Validated living worldwide supercentenarians. Rejuvenation Res.

[19] Rasmussen SH, Thinggaard M, Højgaard MB, Jeune B, Christensen K, Andersen-Ranberg K (2018). Improvement in activities of daily living among Danish centenarians?-a comparative study of two centenarian cohorts born 20 years apart. J Gerontol A Biol Sci Med Sci.

[20] Arai Y, Sasaki T, Hirose N (2017). Demographic, phenotypic, and genetic characteristics of centenarians in Okinawa and Honshu, Japan: part 2 Honshu, Japan. Mech Ageing Dev.

[21] Frisoni GB, Louhija J, Geroldi C, Trabucchi M (2001). Longevity and the epsilon2 allele of apolipoprotein E: the Finnish Centenarians Study. J Gerontol A Biol Sci Med Sci.

[22] ChatGPT. OpenAI.

